# Dysregulation of Expression of Nicotinic Acetylcholine Receptors and Ly6/uPAR Proteins in the Cingulate Gyrus of the Cerebral Cortex of Patients with Alzheimer’s Disease

**DOI:** 10.32607/actanaturae.27904

**Published:** 2026

**Authors:** M. L. Bychkov, A. G. Ochneva, V. M. Ushakova, A. Yu. Morozova, Ya. A. Zorkina, O. V. Abramova, G. P. Kostyuk, M. P. Kirpichnikov, A. S. Paramonov, E. N. Lyukmanova

**Affiliations:** Shemyakin–Ovchinnikov Institute of Bioorganic Chemistry, Russian Academy of Sciences, Moscow, 117997 Russia; Psychiatric Clinical Hospital No. 1 named after N. A. Alekseev, Moscow Department of Healthcare, Moscow, 115191 Russia; Serbsky National Medical Research Center of Psychiatry and Narcology of the Ministry of Health of the Russian Federation, Moscow, 119034 Russia; Faculty of Psychology, Lomonosov Moscow State University, Moscow, 119234 Russia; Russian Biotechnology University (Rosbiotech), Moscow, 125080 Russia; Department of Biology, Lomonosov Moscow State University, Moscow, 119234 Russia; Moscow Center for Advanced Research, Moscow, 123592 Russia; Department of Biology, Shenzhen MSU-BIT University, Guangdong Province, Shenzhen, 518172 China

**Keywords:** Alzheimer’s disease, neurodegeneration, Ly6/uPAR, three-finger proteins, nicotinic acetylcholine receptor, Lynx1, Lynx2

## Abstract

Alzheimer’s disease (AD) is a common neurodegenerative disorder associated with the dysfunction of nicotinic acetylcholine receptors (nAChRs). The function of nAChRs is normally regulated by endogenous ligands, such as acetylcholine and three-finger proteins belonging to the Ly6/uPAR family. However, information on the involvement of these proteins in the pathogenesis of AD is lacking. In this study, by using a bioinformatic analysis, we identified increased mRNA levels of Ly6/uPAR proteins (Lynx1, Lynx2, SLURP-1, Ly6G6E, etc.) in the entorhinal, temporal, and frontal cortex in AD patients with neurodegenerative symptoms, as well as an increase in the mRNA level of Ly6E in the hippocampus in patients with asymptomatic AD. Furthermore, in the cortex and cerebellum, we identified multidirectional changes in the mRNA expression of different nAChR subunits and regulators of inflammation (CD59, CD177, Lypd2, Lypd4, and Lypd8) and metabolism (GPIHBP and Ly6D), also belonging to the Ly6/uPAR protein family. By using immunostaining and Western blotting, we confirmed increased protein levels of Lynx1, Lynx2, and the β2 nAChR subunit in the cingulate gyrus adjacent to the entorhinal cortex in patients with AD, as well as decreased protein levels of the α4 and α7 nAChR subunits. These findings point to complex dysregulation of the cholinergic system in the AD brain at the expression levels both of the individual nAChR subtypes and its natural modulators belonging to the Ly6/uPAR family.

## INTRODUCTION

Alzheimer’s disease (AD) is a neurodegenerative disorder characterized by the accumulation of parenchymal β-amyloid and aggregates of the hyperphosphorylated tau protein, causing a disruption of cholinergic, GABAergic, and glutamatergic neurotransmission and ultimately resulting in the death of neuronal and glial cells in the brain [1, 2, 3, 4]. The key regulators of the cholinergic system function are nicotinic acetylcholine receptors (nAChRs), – the pentameric ligand-gated ion channels activated by acetylcholine or nicotine and involved in the regulation of synaptic transmission, neuroplasticity, and memory [5, 6]. The most abundant nAChRs in the brain are heteropentameric nAChRs consisting of α4 and β2 subunits (α4β2 nAChRs) and homopentameric α7 nAChRs [5, 7]. There are several mechanisms of nAChR function regulation: at the levels of transcription, translation, folding, post-translational modification, and traffick of subunits to the plasma membrane, control of the nAChR assembly and stoichiometry, and interaction with various endogenous neuromodulators [5, 8, 9, 10, 11, 12, 13]. Three-finger proteins belonging to the Ly6/uPAR family are among these modulators of the nAChR function [14]. The mechanism of action of the GPI-anchored protein Lynx1, an allosteric neuromodulator of α7, α4β2, and α3β2 nAChRs, has been investigated most thoroughly [15]. Lynx1 is believed to prevent hyperactivation of nAChRs, thus regulating motor and olfactory memory, neuronal plasticity, and other cognitive processes [16, 17, 18, 19]. Reduced Lynx1 expression in the frontal cortex and cerebellum has been demonstrated in mouse models of AD [20, 21]. Lynx2 is another Ly6/uPAR family member which also modulates nAChRs and regulates anxiety in mice [16]. However, no data on the expression and potential role of these proteins in the pathogenesis of AD in humans are currently available.

In this study, we analyzed the changes in the mRNA expression of Ly6/uPAR family proteins and nAChR subunits across multiple brain regions in AD patients using the data from the Gene Expression Omnibus database and confirmed the revealed pattern for the α7, α4, and β2 nAChR subunits, as well as for Lynx1 and Lynx2, in the cingulate gyrus adjacent to the entorhinal cortex of AD patients.

## EXPERIMENTAL

The mRNA expression of Ly6/uPAR proteins across different brain regions of AD patients was analyzed using the data from the Gene Expression Omnibus database (the datasets GSE36980 for the hippocampus and GSE118553 for different cortical regions). The analysis was conducted using the Geo2R tool [22].

Postmortem tissues of the cingulate gyrus of the cerebral cortex from patients with and without AD (four patients per group) were acquired from the Psychiatric Clinical Hospital No. 1, Moscow Department of Healthcare (the biospecimen collection procedure was approved by the institutional ethics committee, Protocol No. 2, 28 October 2020). The tissues were frozen on dry ice and stored at −80°C until analysis. For immunostaining, they were fixed for 20 min in a cold 1 : 1 acetone–methanol mixture, subjected to epitope retrieval (Declere buffer, Cell Marque, Canada; 20 min, 90°C), and cut into 10 µm sections on an Erpedia cryotome (Thermo Fisher, USA). The sections were stained sequentially several times. First, the sections were incubated for 16 h with primary mouse antibodies against the α7 nAChR subunit (ABIN5611363, Antibodies Online, Germany) and rabbit antibodies against the α4 nAChR subunit (ABIN5013334, Antibodies Online), washed three times in phosphate-buffered saline, and incubated with secondary anti-mouse antibodies (Per-CP, 407111, BioLegend, USA) and anti-rabbit antibodies (AlexaFluor 647, Jackson Immunoresearch, USA), respectively, for 3 h. Sections were then washed three times in PBS and incubated for 16 h with primary mouse antibodies against the β2 nAChR subunit (ABIN561131, Antibodies Online), anti-Lynx1 rabbit antibodies (ab125035, Abcam, UK), and anti-Lynx2 sheep antibodies (AF6855, R&D Systems, USA). The sections were then washed three times in PBS and incubated with secondary anti-mouse (TRITC, 715-025-151, Jackson Immunoresearch), anti-rabbit (AlexaFluor 488, 611-545-215, Jackson Immunoresearch), and anti-sheep (DyLight 405, 713-475-003, Jackson Immunoresearch) antibodies, respectively, for 3 h. The detailed data on the antibodies, buffers, and incubation times are provided in the *Supplementary Materials* (*Table S1*). The sections were then embedded in Mowiol-DABCO (Sigma-Aldrich, USA) on a coverslip and analyzed using a Carl Zeiss LSM710 confocal microscope with a ×63 (1.4) objective (Carl Zeiss, Germany). Each fluorophore was excited and recorded in a separate image acquisition track; the wavelength ranges on a detector were set so as to prevent fluorescence bleed-through: DyLight 405 (excitation, 405 nm; detection, 420–460 nm), AlexaFluor 488 (excitation, 488 nm; detection, 510–550 nm), TRITC (excitation, 561 nm; detection, 568–588 nm), Per-CP (excitation, 514 nm; detection, 650–700 nm), and AlexaFluor 647 (excitation, 633 nm; detection, 650–700 nm). Z-stacks 15 µm thick were acquired. The lipophilic dye DiI (1 µM in acetone) was then added without removing the coverslip from the objective; after 1 h, stained nerve endings were imaged (excitation 514 nm, detection 520–600 nm).

The images were analyzed using the ImageJ 1.5f software (NIH, Bethesda, USA). The image was filtered (unsharp mask, σ = 25, threshold, percentile, 90% + standard deviation of pixel intensity). The number of clusters of the studied proteins (find maxima), cluster fluorescence intensity (measure), and the number of clusters of different diameters were calculated by setting the threshold cluster diameter using the “analyze particles” option. Clusters with a diameter > 0.1 µm were selected. Therefore, within the context of this work, “cluster” denotes a “dot” stained by antibodies specific to the studied protein.

Western blotting was performed according to the procedure described in [20]. Cingulate gyrus tissues from patients were lysed in RIPA buffer containing SIGMAFAST protease inhibitors (Sigma-Aldrich); proteins were separated by polyacrylamide gel electrophoresis (PAGE) and transferred to nitrocellulose membranes. After the incubation with primary mouse antibodies against the α7 nAChR subunit (ABIN5611363, Antibodies Online) and β2 nAChR subunit (ABIN561131, Antibodies Online), primary rabbit antibodies against the α4 nAChR subunit (ABIN5013334, Antibodies Online) and Lynx1 (ab125035, Abcam), as well as primary anti-Lynx2 sheep antibodies (AF6855, R&D Systems) and secondary anti-mouse (715-035-150, Jackson Immunoresearch), anti-rabbit (711-035-152, Jackson Immunoresearch), and anti-sheep (713-035-003, Jackson Immunoresearch) antibodies, the HRP signal was captured using the ECL substrate (Bio-Rad, USA) and a LAS500 imager (GE Healthcare, USA). The detailed information on the antibodies, buffers, and incubation times are provided in the *Supplementary Materials* (*Table S1*). After protein expression had been detected, the membranes were stained for β-actin (GB11001, ServiceBio, China) using the procedure described earlier.

Statistical analysis of the data was performed using the GraphPad Prism 10.4.0 software (GraphPad Software, USA).

## RESULTS AND DISCUSSION

Cognitive impairment in AD, which is associated with cholinergic system dysfunction, is often accompanied by alterations in gene expression [5, 23]. Analysis of the Gene Expression Omnibus database revealed multidirectional changes in the mRNA expression of Ly6/uPAR proteins across different brain regions in patients with asymptomatic AD (*Fig. 1A*). However, upregulated mRNA expression of Ly6/uPAR proteins was mostly observed in AD patients with neurodegeneration symptoms (*Fig. 1A*). Changes in the mRNA expression of Ly6/uPAR proteins in AD patients may indicate their involvement in neuroinflammation (as it is characteristic for CD59, – the regulator of the complement system; the inflammation inhibitor Lypd8; and the modulator of immune cell migration CD177), triglyceride metabolism (GPIHBP mediates the breakdown of triglycerides in the bloodstream), cellular stress (Ly6D stimulates inflammation and lipid accumulation in hepatocytes), and regulation of the nAChR function (Lynx1 and Ly6E). Interestingly, *LYNX1* expression is also significantly upregulated in the cortex in patients with asymptomatic AD (*Fig. 1A*). This fact contradicts the earlier data obtained using the AD model in 3xTg mice with overexpression of the mutant β-amyloid precursor protein and tau protein, in which Lynx1 expression in the frontal cortex appeared reduced at the protein level [21]. Meanwhile, the decrease (although not significant) in the cerebellar *LYNX1* expression at the asymptomatic stage of AD is consistent with the previously observed decline in the cerebellar expression of this neuromodulator in 2xTg mice with overexpression of the mutant β-amyloid precursor protein but with a normal expression level of the tau protein [20].

**Fig. 1:**
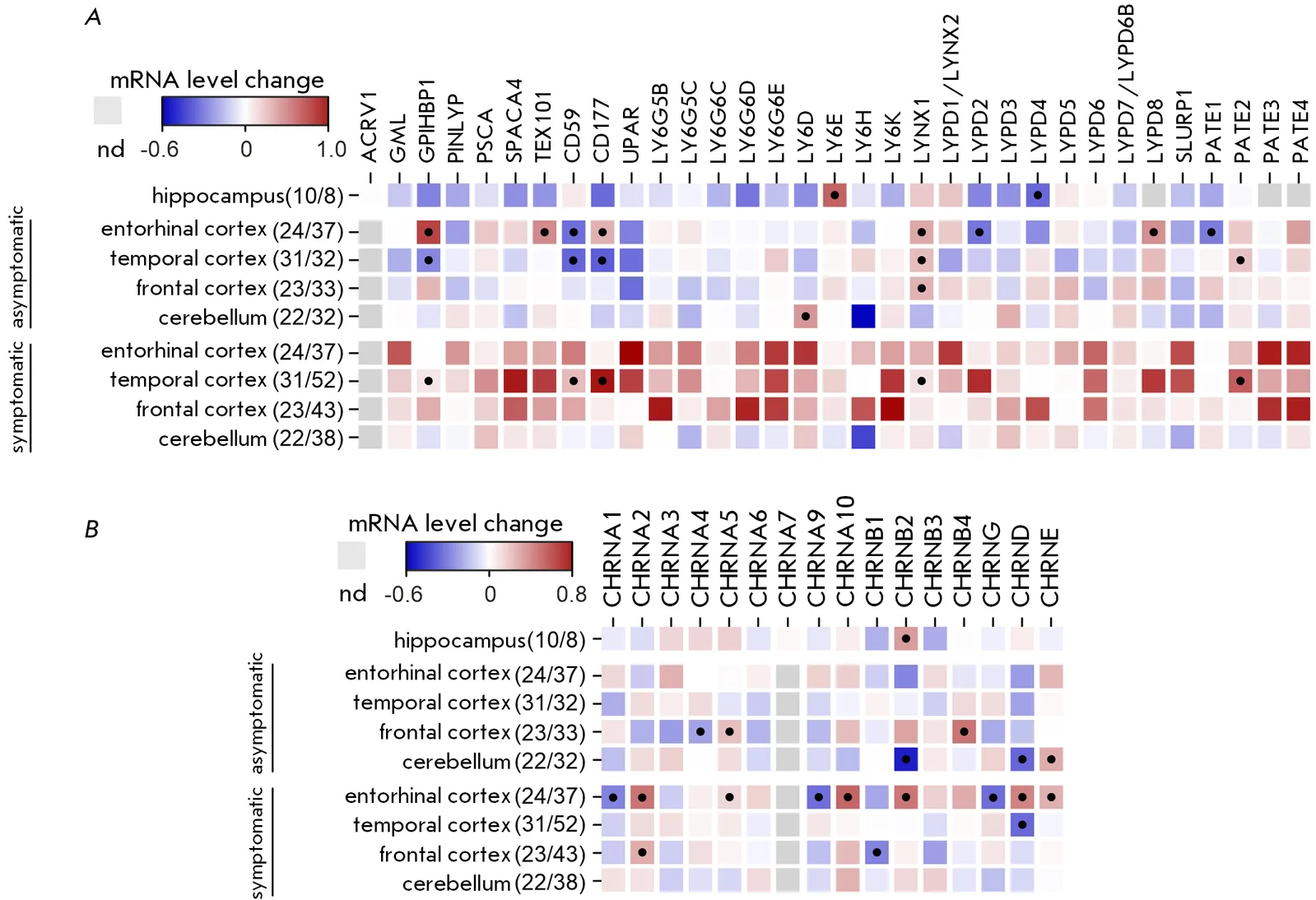
mRNA expression of Ly6/uPAR proteins (*A*) and various nAChR subunits (*B*) in the human AD brain according to the Gene Expression Omnibus database. The data were analyzed using the Geo2R graphical interface and then downloaded for statistical analysis using the GraphPad Prism software. The number of healthy individuals and AD patients is shown in the figure in parentheses (*n* individuals without AD / *n* patients with AD). Significant changes compared to healthy donors according to the two-sided Mann–Whitney U test with the Holm–Sidak *post-hoc* test are shown as black dots (*n* = 8–52). n.d. – no data on the level of this mRNA are available

The bioinformatic analysis also revealed multidirectional changes in the mRNA expression of nAChR subunits; the greatest number of significant changes in the entorhinal cortex was detected in patients with symptomatic AD (*Fig. 1B*). Upregulated mRNA expression was observed for the α2, α5, α10, β2, δ, and ε nAChR subunits, whereas the mRNA expression of the α1, α9, and γ nAChR subunits was downregulated (*Fig. 1B*). In the frontal cortex, mRNA expression of α2 nAChR was also upregulated, while mRNA expression of β1 nAChR was downregulated (*Fig. 1B*). No changes in the expression of the *CHRNA7* gene encoding the α7 nAChR subunit were detected in the brain regions analyzed.

Differences in the altered patterns of expression of the same genes at different stages of AD may be a sign that processes occurring in the brain at the early and late stages of the disease vary. For example, the tau protein and β-amyloid oligomers accumulate in neurons that remain functional at the asymptomatic early stage of AD; in this case, the compensatory mechanisms (e.g., isomorphic astrogliosis) are triggered and microglia is activated [24]. The symptomatic stage of AD is characterized by neuronal death, the formation of an astrocytic “barrier” isolating the damaged brain region (anisomorphic astrogliosis and accumulation of metabolically hypoactive astrocytes), and a more pronounced neuroinflammation [24, 25, 26]. The multidirectional changes in *GPIHBP1* and *CD177* expression in the entorhinal and temporal cortex at the early stage of AD may be indication of the predominance of distinct regulatory mechanisms characteristic of these brain regions, which requires further research.

Overall, the bioinformatic analysis data point toward potential disruption in the nAChR function in AD, which, among other factors, associates with the impaired expression of its neuromodulators belonging to the Ly6/uPAR family. In order to test our findings at the protein level, we used immunostaining to compare the abundance of certain studied proteins in the tissues of the cingulate gyrus adjacent to the entorhinal cortex from patients with and without AD. Immunostaining revealed a diffuse pattern of distribution of the α4 and β2 nAChR subunits, and Lynx2, within the cingulate gyrus tissue, while the expression levels of α7 nAChR and Lynx1 tracked more closely the nerve endings stained with DiI (*Fig. 2A*). It is fair to assume that the α4 and β2 nAChR subunits, as well as Lynx2, preferentially localize in the nonterminal neuronal endings, while the α7 nAChR subunit and Lynx1 are preferentially expressed in the dendrites. However, this hypothesis needs additional testing.

**Fig. 2:**
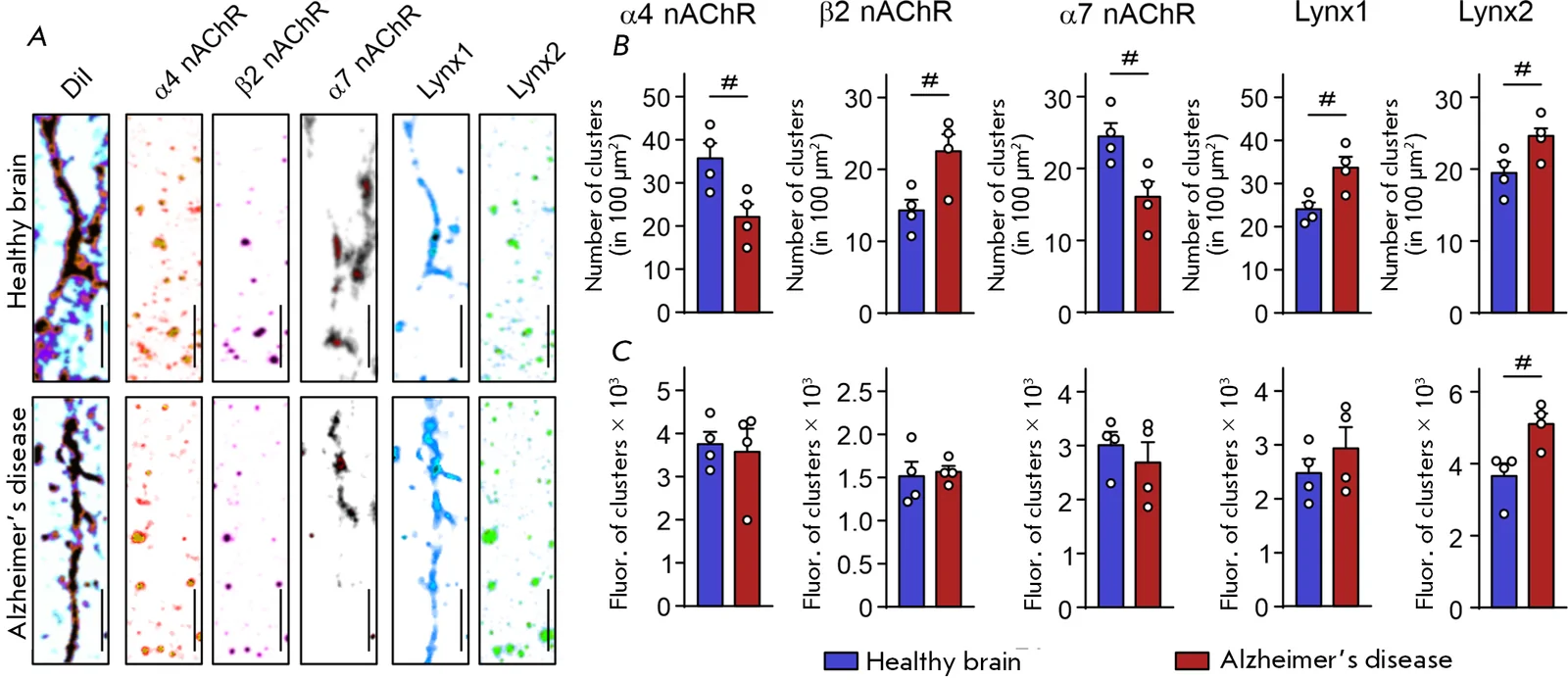
Comparative analysis of the expression of the Lynx1 and Lynx2 proteins and α4, β2, and α7 nAChR subunits in the cingulate gyrus of the cerebral cortex of patients with and without AD. (*A*) Representative images of nerve endings in the cingulate gyrus of the cerebral cortex of healthy donors and AD patients visualized with DiI and clusters of the Lynx1, Lynx2, α4, α7, and β2 nAChR subunits, scale bar = 15 μm. (*B*) Analysis of the number of clusters (size greater than 0.1 μm) of the studied proteins. Data are presented as the number of clusters in 100 μm^2^ ± SEM (*n* = 4). # (*p* < 0.05) indicates a significant difference between the data groups according to the two-tailed Mann–Whitney U-test. (*C*) Analysis of the fluorescence intensity of the clusters (size greater than 0.1 µm) of the studied proteins. Data are presented as the mean fluorescence intensity of all clusters ± SEM (*n* = 4). # (*p* < 0.05) indicates a significant difference between the data groups according to the two-tailed Mann–Whitney U-test

A statistically significant decrease in the number of clusters of α4 and α7 nAChR subunits and rise in the number of clasters of Lynx1, Lynx2, and the β2 nAChR subunit were revealed in the AD tissues (*Fig. 2B*). In AD tissues, increased fluorescence intensity was observed only for the Lynx2 clusters (*Fig. 2C*). An analysis of the cluster diameter demonstrated that AD tissues have a larger number of small clusters (diameter, 0.1 and 0.3 µm) of the α4 nAChR subunit, while α7 nAChR, Lynx1, and Lynx2 are characterized by an increased number of clusters with both small (0.3, 0.6 and 0.9 µm) and larger (≥ 10 µm) diameters (*Fig. 3*). Meanwhile, no changes in the diameter of β2 nAChR subunit clusters were detected (*Fig. 3*). Hence, the decline in the number of α4 nAChR clusters was accompanied by a reduction of their diameter, while the increase in the number of Lynx1 and Lynx2 clusters was accompanied by the emergence of both small and larger aggregates of these proteins. Changes in the cluster diameter *per se* are unlikely to be indication of alterations in nAChR activity but may suggest that the emergence of small clusters of the α4 nAChR subunit is associated with a shift from the low-sensitivity stoichiometry of α4β2 nAChR pentamers ((α4)_3_(β2)_2_) to the high-sensitivity one ((α4)_2_(β2)_3_) to compensate for the lack of the receptor. Furthermore, the elevated content of larger α7 nAChR clusters can be related to aggregation of this type of the receptor caused by its direct interaction with toxic β-amyloid oligomers [27]. It is worth noting that nAChRs can form complexes with other cell surface receptors engaged in synaptic plasticity. Thus, in epithelial cells, α7-nAChR forms complexes with the receptor kinases EGFR and PDGFRβ [28] engaged in long-term potentiation and neurite growth [29, 30], while the β2 nAChR subunit interacts with VEGFR2, which maintains neuronal viability under oxidative stress conditions [31]. Therefore, the revealed changes in the diameter of nAChR subunit clusters, as well as of clusters of their modulators Lynx1 and Lynx2, may suggest that the modulation of the formation of these supramolecular signaling complexes has been altered.

**Fig. 3:**
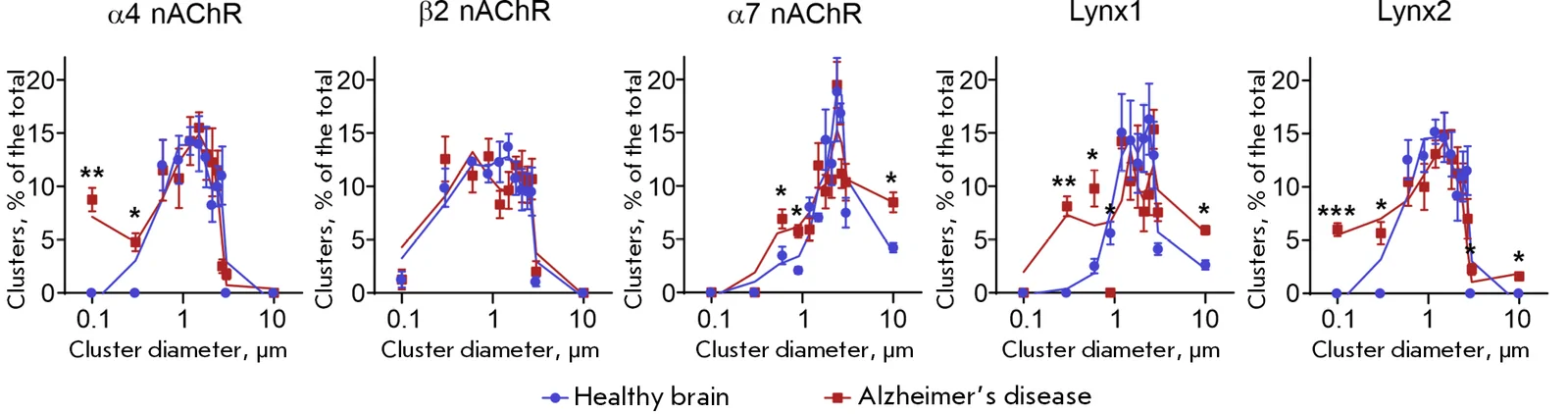
Analysis of the distribution of clusters of different diameters for the studied proteins in the range from 0.1 to 10 µm (single clusters of larger diameters for α7 nAChR and Lynx1 were assigned to the “10 µm” group). Data are presented as a percentage of clusters of different diameters from the total number ± SEM (*n* = 4). *(*p* < 0.05), **(*p* < 0.01), and ***(*p* < 0.001) indicate a significant difference between the data groups (patients with and without AD) according to a two-tailed t-test with the Holm–Šidák *post-hoc* test

Western blotting revealed a statistically significant decrease in the protein levels of the α4 and α7 nAChR subunits, as well as elevated levels of the Lynx1 and Lynx2 proteins in the cingulate gyrus of the cerebral cortex in the tissues of AD patients (*Fig. 4A*,*B*). Although being consistent with the bioinformatic analysis data (*Fig. 1B*), the apparent increase in β2 nAChR expression was not statistically significant (*Fig. 4A,B*). The bioinformatic analysis revealed no changes in the mRNA expression of α7 nAChR, although the protein level of this receptor had declined significantly (*Fig. 4B*). Furthermore, the intensity of two bands of Lynx2 proteins of different molecular weights was increased in the AD tissues; these bands can potentially correspond to the GPI-anchored and secreted protein forms, as demonstrated previously for the Lynx1 protein [20, 21] or suggest that Lynx2 may form complexes with other proteins in the human brain.

**Fig. 4:**
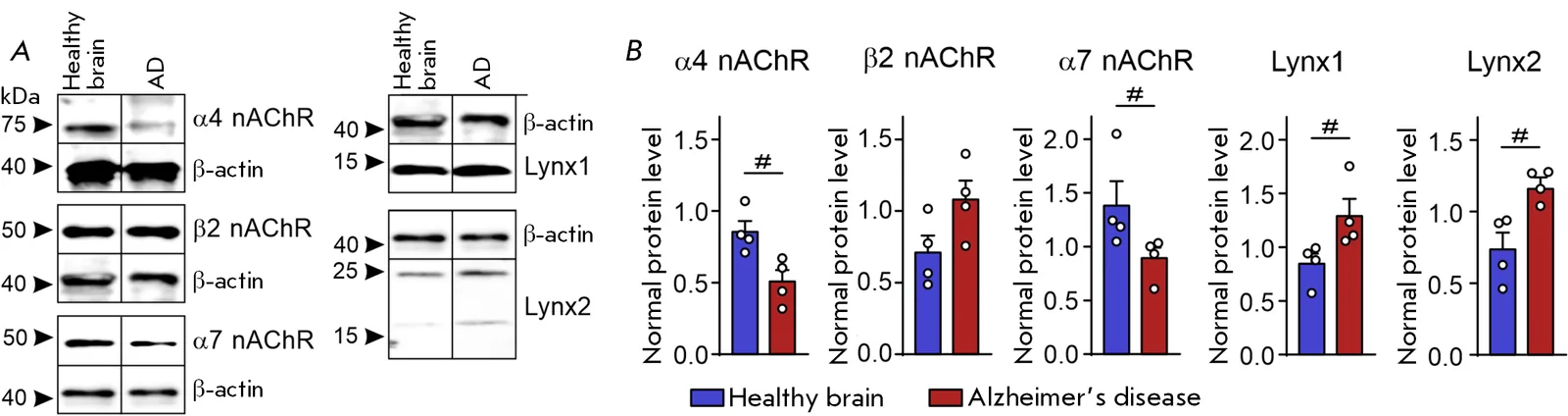
Western blot analysis of the expression of the Lynx1 and Lynx2 proteins and α4, β2, and α7 nAChR subunits in the cingulate gyrus of the cerebral cortex of patients with and without AD. (*A*) Representative protein bands (full membranes are shown in the *Supplementary Materials*, *Fig. S1*). (*B*) Protein expression analysis. Data are presented as the level of the target protein normalized to the level of β-actin ± SEM (*n* = 4). # (*p* < 0.05) indicates a significant difference between the data groups according to the two-tailed Mann–Whitney U-test

The upregulation of Lynx1 expression both at the gene and protein levels in the AD tissues (*Figs. 1* and *4*) emphasize the fact that the mouse models of AD, in which decreased Lynx1 protein levels were earlier detected in the frontal cortex [21] and cerebellum [20], are inconsistent with the actual pathogenesis of this disease in humans. This inconsistency may be indication that the mechanisms of signal transduction, metabolism regulation, neuroinflammation, and the compensatory processes occurring in the brain under normal and pathological conditions differ in humans and mice.

The Lynx1 protein modulates the function of α4β2- and α7-nAChRs via several mechanisms, whose key is the modulation of receptor activity at the plasma membrane: the Lynx1 protein allosterically modulates receptor function, thus preventing hyperactivation [17, 32]. Furthermore, Lynx1 is involved in gene transcription regulation in the hippocampal neurons [33] and promotes the assembly of low-sensitive ((α4)_3_(β2)_2_)-nAChR pentamers in the endoplasmic reticulum [34]. Normally, Lynx1 constrains synaptic plasticity [18, 19], thus preventing hyperactivation of the cholinergic system. The Lynx2 protein allosterically inhibits the function of α4β2- and α7-nAChRs by enhancing channel desensitization [35, 36]. Furthermore, Lynx2 regulates the α4β2-nAChR trafficking to the plasma membrane surface [36]. Modulation of the cholinergic system through Lynx2 is required for the regulation of anxiety and social interactions [10]. Hence, Lynx1 and Lynx2 normally act as allosteric modulators of α4β2- and α7-nAChRs, modulating both the function and expression of these receptors on the membrane surface of neurons.

Expression of functionally active α4β2- and α7-nAChRs in the human AD brain is downregulated [37, 38], which is consistent with our data (*Fig. 4B*). We have revealed that this downregulation is accompanied by upregulated expression of Lynx1 and Lynx2 in the brain (*Figs. 1* and *4*), which may cause dysfunction of the cholinergic system in AD. In fact, simultaneous suppression of the expression of the α4 and α7 nAChR subunits and increased control over the function of the receptors composed of these subunits due to upregulated Lynx1 and Lynx2 expression take place. This regulatory receptor/modulator imbalance may mediate neurodegeneration in AD.

## CONCLUSION

In this study, we analyzed for the first time the expression of the Lynx1 and Lynx2 neuromodulators in brain tissues derived from AD patients. Along with upregulated expression of both proteins, we observed multidirectional changes in the mRNA expression of various nAChR subunits and other Ly6/uPAR proteins involved in the regulation of the cholinergic system, neuroinflammation, and metabolism. The downregulated expression of the key cholinergic receptors consisting of the α4 and α7 subunits and the simultaneously upregulated expression of their neuromodulators may cause the dysfunction of the cholinergic system observed in AD.

## Abbreviations

AD: Alzheimer’s disease
nAChR: nicotinic acetylcholine receptor
PBS: phosphate-buffered saline
